# The where of bodily awareness

**DOI:** 10.1007/s11229-019-02171-3

**Published:** 2019-04-08

**Authors:** Alisa Mandrigin

**Affiliations:** 1 Division of Psychology, University of Stirling, Stirling, FK9 4LA UK; 2 Division of Law and Philosophy, University of Stirling, Stirling, FK9 4LA UK

**Keywords:** Bodily awareness, Spatial content, Proprioception, Touch, Body representation, Perception

## Abstract

In bodily awareness body parts are felt to occupy locations relative to the rest of the body. Bodily sensations are felt to be, in Brian O’Shaughnessy’s terms ‘in-a-certain-body-part-at-a-position-in-body-relative-physical-space’. In this paper I put forward a dispositional account of the structure of the spatial content of bodily awareness, which takes inspiration from Gareth Evans’s account of egocentric spatial content in *The Varieties of Reference* (1982). On the Dispositional View, bodily awareness experiences have spatial content in virtue of a set of connections having been established between somatosensory and proprioceptive inputs on the one hand, and motor outputs on the other hand. This kind of account, according to which spatial content depends constitutively on bodily action, has been challenged by a set of neurological cases and behavioural studies on healthy subjects. The evidence has been used to motivate a functional distinction between two kinds of body representation: representations for perception and representations for action. I review and assess some of the main sources of evidence for this distinction, arguing that the evidence presents a challenge to the dispositional view only if we accept the unjustified assumption that differences in task performance can only be explained in terms of a difference in representation. I close by proposing, and offering some empirical support for, an alternative explanation of the empirical results. The availability of the alternative explanation means that further work is needed to establish whether or not there is any challenge to the Dispositional View.

## The spatial structure of bodily awareness

While we can gain information about the body through the exteroceptive senses, we also receive various kinds of information about the current state of our bodies ‘from within’. This includes information from cutaneous receptors about pressure, temperature and friction; information from receptors in the joints and from skin-stretch about the dynamic and static disposition of the limbs; and information from the muscles about effort and muscular fatigue.^1^ Not all the information we receive through these body senses gives rise to conscious experience. Still, we feel bodily sensations such as pains and tickles, tactile sensations arising from bodily contact, and we can be aware of the position of our limbs and their movement. I will use ‘bodily awareness’ to pick out conscious experiences of properties of the body arising from the processing of information generated ‘from the inside’.^2^

In feeling pains, tickles, and tactile sensations, we feel a part or several parts of our body to be some way or other: to hurt, or to itch, and so on. As M.G.F. Martin says, “the qualities that characterise the experience qualify the part of the body that one is aware of” (1995, p. 268).^3^ Still, in claiming that the object of an episode of bodily awareness is a body part that is felt to be a certain way—that is, for example, felt to itch or ache—we should not lose sight of the fact that the body itself is as much the object of perception. It would be a mistake, I think, to say that structure plays no role in determining what is perceived in bodily awareness, and a mistake to say that in bodily awareness we don’t perceive the body to have structure. This is because body parts are not normally presented in bodily awareness as being in thin air, as being located at points in space. To claim this would be to distort or misrepresent the phenomenology of bodily awareness. For parts of the body are presented to us in bodily awareness as located relative to the location of the other parts of the body, and thereby as being within the bounds of the body as a whole (Martin 1995; Brewer 1995). Brian O’Shaughnessy says that:

The basic ‘given’ is, not just feeling, not just feeling-in-a-certain-body-part, but *feeling*-*in*-*a*-*certain*-*body*-*part*-*at*-*a*-*position*-*in*-*body*-*relative*-*physical*-*space*; and so, also, certain-body-part-at-a-position-in-body-relative-physical-space: the latter being disclosed along with and via the former *and* the former being disclosed along with and via the latter. (O’Shaughnessy 1980, p. 165)

When I feel a pain in my neck, my experience is as of my neck hurting, where the body part that is felt to hurt—the neck—is felt to occupy a location relative to the rest of the body. Bermúdez labels this the ‘connectedness’ of bodily awareness: “the spatial location of a bodily event is experienced relative to the disposition of the body as a whole” (Bermúdez 2017, p. 126). A body part that is felt to hurt or itch or to be warm feels to be at a position in body-relative space.

We also experience our posture and the disposition of our limbs. I can be aware that my legs are crossed or that my arms are straight and hanging by the side of my torso. In such cases, I am aware of the location of my limbs, my head, my torso, and so on. And, as with bodily sensations, our awareness of our posture seems to be body-relative. When I experience my legs as crossed, I am aware of the location of my legs relative to one another and to other parts of my body. When I have an experience of my arms as hanging by my side, I am aware of the location of both of my arms relative to my torso, and so on. What it is, then, to be aware of one’s posture, is to be aware of the position of the parts of one’s body relative to the other parts of the body.

The spatial content of bodily awareness is integral to our conscious experiences of our bodies ‘from the inside’. It presents us with a structured relation between body parts and whole, such that sensations and the disposition of one’s limbs are presented as occurring within body-relative space. How, though, might we account for this structuring of the spatial content of bodily awareness?

## A dispositional theory of the spatial content of bodily awareness

Sensory inputs carry or encode information about location in that they “belong to a range of inputs which vary systematically with some spatial facts” (Evans 1982, p. 154). This is no less true of the body senses, than it is of, say, vision and audition. But the fact that sensory inputs encapsulate such information offers no explanation of how it is that we are able to see objects as occupying locations in egocentric space, or feel body parts as occupying locations in body-relative space. How is it that these sensory inputs, which count as conveying spatial information only in so far as they stand in some kind of systematic relation to other possible sensory inputs, come to have spatial significance for the perceiving subject? According to Gareth Evans, “we must say that having spatially significant perceptual information consists at least partly in being disposed to do various things” (1982, p. 155). For Evans:

Egocentric spatial terms and the terms in which the content of our spatial experiences would be formulated, and those in which our immediate behavioural plans would be expressed. The duality is no coincidence: an egocentric space can exist only for an animal in which a complex network of connections exists between perceptual input and behavioural output. A perceptual input—even if, in some loose sense, it encapsulates spatial information (because it belongs to a range of inputs which vary systematically with some spatial facts)—cannot have a spatial significance for an organism except in so far as it has a place in such a complex network of input–output connections. (1982, p. 154)

If we apply this approach to the spatial content of bodily awareness, we find ourselves with the following picture.^4^ What is required for a perceiver to have a grasp of bodily spatial contents—what it is for us to perceive body parts as occupying locations in body-relative space—is that a complex set of connections have been established between somatosensory and proprioceptive inputs on the one hand, and motor outputs on the other. For one’s bodily awareness experiences to have spatial content, a complex network of connections between somatosensory and proprioceptive inputs and motor outputs must be in place. So, experience of location in bodily awareness is constitutively tied to having dispositions to engage in bodily actions directed towards that bodily location. I’ll call this the Dispositional View of the spatial content of conscious bodily awareness.

With the Dispositional View, we adopt a view according to which the spatial content of conscious bodily awareness depends constitutively on bodily action. A number of philosophers have recommended this kind of approach to the spatial content of bodily awareness. For example, in this defence of what he calls common sense anti-Cartesianism, Bill Brewer suggests that, “the intrinsic spatial content of normal bodily awareness is given directly in terms of practical knowledge of how to act in connection with the bodily locations involved” (1995, p. 302).^5^ The view that Brewer seems to be advocating here is one according to which locations are represented in terms of the movements of the body that are required for the body part in question to be reached or grasped. By contrast, the Dispositional View doesn’t reduce spatial content to motor behaviour. The spatial content of one’s bodily awareness experience is not to be identified with the specification for a motor behaviour or set of motor behaviours that would allow the perceiver to reach to the bodily location. Rather, on the Dispositional View, one’s experience will have spatial content only if, given the appropriate beliefs and desires, one is disposed to engage in actions directed toward that bodily location. That is, it will have spatial content only if a complex set of connections between sensory inputs and motor outputs is in place, such that the sensory input, in conjunction with an appropriate set of cognitive states that together act to motivate a response, will dispose the perceiver to respond with a particular set of motor behaviours (Grush 2007; Briscoe 2014).

An account that draws a constitutive connection between perception and action is appealing for a number of reasons. First, it offers a straightforward explanation of why it is that our bodily awareness experiences seem to facilitate immediate and unmediated action (Evans 1982, pp. 155–156). When I feel a mosquito bite on my leg, I don’t need to think about how I should move my hand in order to scratch the itch that I feel (de Vignemont 2011). Our motor actions in response to bodily sensation seem to be automatic: I don’t have to engage in any reasoning or deliberation about how I should move my arm and hand in order to scratch the spot at which I feel the itch. As Brewer notes:

[…] it is impossible to erase the immediate inclination to act in connection with the particular location of a bodily sensation from our conception of the epistemological given in bodily awareness. When I feel a sharp pain in the back of my left hand or an itch on the end of my nose, the appropriateness of action concerning these actual bodily locations is written into the very nature of the experience itself […]. (Brewer 1995, p. 298)

The Dispositional View of the spatial content of bodily awareness is well placed to explain this, since it recommends that having spatial content is intimately connected with having dispositions to perform appropriate bodily actions. Bodily awareness experiences facilitate immediate action because they have their spatial content in virtue of established connections between sensory input and motor outputs. To perceive a body part at a location in body-relative space is to have an experience whose content is poised to guide one’s bodily actions in relation to the body part (Briscoe 2014, p. 208).

Another reason, adduced by Robert Briscoe in his development of Evans’s account of egocentric spatial content (2014), concerns the biological function of perception. With the focus on vision, Briscoe says that:

From a biological or evolutionary standpoint, it is reasonable to think that vision is *for* action, that its preeminent biological function is to adapt an animal’s bodily movements to the properties of the environment that it inhabits. (Briscoe 2014, p. 202)

The same seems true of conscious bodily awareness. It seems reasonable to think that the primary biological function of conscious bodily awareness is to adapt the perceiver’s bodily movements to be responsive to the properties that obtain and the events that occur within particular parts of her body.

However, a body of empirical work seems to indicate the possibility of dissociations between conscious bodily awareness and action. As such, it looks as though it poses a threat to the Dispositional View, and any view that claims there is a constitutive link between the spatial content of bodily awareness and bodily action.

## The empirical challenge

### Dissociations between perception and action in patients with neurological conditions

Due to central deafferentation of the limb, patients with numbsense report that they do not have any tactile or proprioceptive experience in affected limbs. When asked to localize tactile stimulation on the affected limb in the absence of vision either verbally or by pointing with the unaffected hand to a diagram of the hand, they are at chance. Yet, when asked to point with the unaffected hand to the site of stimulation on the affected limb, they can do so reliably (Paillard et al. 1983; de Vignemont 2011; Wong 2015).

We can contrast these patients with patients who are peripherally deafferented. Some patients, who have extensive loss of tactile sensation and proprioception due to destruction of the nerves, have been able to learn to use visual feedback to control bodily movement in the affected limbs. And, while they now lack touch and proprioception, pain and temperature sensations have been preserved. When a nociceptive or thermal stimulus is applied to an affected hand in the absence of vision, peripherally deafferentated patients are unable to point to the locus of stimulation with the unstimulated hand. Yet, despite this, they are able to make a verbal report about the location of the stimulation, and, when vision of the unstimulated hand is available to them, point to the location of stimulation on a diagram of the hand.

We find a similar pattern of performance in patients KE and JO, both of whom have suffered a stroke causing neurological damage, but leaving somatosensory detection in place (Anema et al. 2009). Both KE and JO are able to detect tactile stimulation. Yet, they exhibit different abilities to localize the touch that they are able to detect when given two different localization tasks. In one task they were asked to point to the location of stimulation on a diagram of their hand; in the other task they were asked to point directly to the location of stimulation on their own hand. Both exhibited errors in pointing in both tasks. But while JO made smaller errors when locating the point of stimulation on her own hand compared to the diagram, KE was better able to locate the point of stimulation on the diagram compared with localization on his own hand (Anema et al. 2009, p. 1619).

### A dissociation between bodily awareness and action in neurologically healthy subjects

In the standard version of the rubber hand illusion (RHI) (Botvinick and Cohen 1998; Tsakaris and Haggard 2005, for example) subjects sit with their own hands in front of them, with one hand hidden from sight. What they see, in place of their own hidden hand, is a rubber hand, which is spatially displaced laterally relative to their own hand. Subjects are then stroked with a paintbrush on their own hidden hand, and this stroking is either synchronous or asynchronous with stroking that they see being applied to the rubber hand.

In the synchronous compared to the asynchronous condition, participants exhibit what is called proprioceptive drift: that is, when asked to locate their hand when both the rubber hand and their actual hand are hidden from view, they mis-locate their own hand in the direction of the rubber hand. Standardly, this is interpreted as indicating that the participant’s bodily awareness experiences have illusory spatial content: after synchronous stroking, participants feel that their hand is located nearer to the location of the rubber hand.

Yet, despite these results, Kammers et al. (2009) found that when they asked participants to point to the stimulated hand with their other hand in the absence of vision, they were able to do so accurately. And, they could point accurately with the stimulated hand to the unstimulated hand. So, their inter-manual pointing responses were not subject to any illusion.

What is more, when asked again to make a judgement about the location of their hand, they still exhibited proprioceptive drift. Even though they had just performed an accurate inter-manual movement, participants’ judgments suggest that they were still experiencing an illusion about the location of their own hand.

### Representations for perception and representations for action

These apparent dissociations between bodily awareness and action seem to mirror those found between conscious visual perception and visually guided action. The latter dissociations have been taken to warrant a distinction in the functional architecture of visual processing, and a number of philosophers have argued that the evidence supporting this Two Visual Systems Hypothesis counts against the claim that there is a constitutive link between visual perception and action (see, for example, Clark 1999, 2001, 2007; see Briscoe 2009 for a rebuttal of these claims). The idea, roughly, is that if visual information for conscious perception is processed along a separate pathway from visual information for action, then the spatial content of the former cannot be constitutively linked to the perceiver’s bodily action.

De Vignemont (2009, 2011, 2018) makes a similar attack on an action-based account of the spatial content of bodily awareness. De Vignemont focuses her attention on what she calls bodily know-how. In her (2011) and (2018), De Vignemont’s target is, in fact, the enactive approach to perception, associated with Hurley (1998) and Noë (2004), as applied to passive touch. According to the enactive approach, to perceive an object as having spatial properties (including location), one must have implicit knowledge of a set of sensorimotor contingencies: the ways that sensory inputs vary as a function of bodily movement. De Vignemont argues that we cannot make sense of an enactive account of passive touch for a number of reasons, one of these being that passive touch can be instantaneous, and so we cannot make sense of there being any sensorimotor expectations associated with it. One’s perceptual experience of a brief tap on the knee cannot be explained in terms of expectations about the ways that sensory input will change as a result of bodily movement: there is no on-going sensory input. De Vignemont counsels that the best option seems to be to switch sensorimotor knowledge for mere motor knowledge, so that “the spatial content of the tactile experience that I was touched on the knee is determined by the procedural knowledge of how to get to the bodily location of the tactile stimulation” (de Vignemont 2011, p. 194), which she labels ‘bodily know-how’.

De Vignemont argues that the empirical work summarised in Sects. 3.1 and 3.2 poses a challenge to this ‘bodily know-how’ view. Take peripheral deafferentation: it looks to be a case in which a painful or thermal sensation is felt at a location on the body, but in which the subject lacks bodily know-how. Failure to point to the location of stimulation indicates that the peripherally deafferented subject lacks bodily know-how: she lacks knowledge of how to get to the location of stimulation. Nevertheless, she experiences painful sensations as being at bodily locations. As such, it looks as though peripheral deafferentation provides evidence that bodily know-how is not required for bodily experiences with spatial content.^6^ Similarly:

KE experiences and reports that he is touched on the hand on a specific location, but he is unable to get to the location of the touch on his own hand. Hence, there is no spatial know-how that could provide the spatial content of his tactile experience, and the tactile spatial content must have a different ground. (de Vignemont 2011, p. 198)

So, de Vignemont argues that KE shows that bodily know-how is not necessary for a subject to have experiences with spatial content. In cases of neurological disorder it’s possible for a patient to have a conscious experience of a tactile sensation with a felt location on which he can report, yet be unable to point to the location of stimulation on his own hand. In KE it looks as though we have a case in which bodily know-how is absent, and yet the patient experiences tactile sensations to have a felt location.

De Vignemont offers a similar diagnosis of performance in the RHI experiment. In this case, participants had the procedural knowledge of how to move one hand to touch the other—they succeeded on the inter-manual reaching task—but all the while they seemed to have a bodily experience with illusory spatial content. De Vignemont concludes that:

in healthy individuals […] the spatial content of tactile and proprioceptive experiences can be dissociated from the spatial information encoded in spatial know-how used to guide reaching and pointing movements, such that one can be inaccurate and not the other, and vice versa. […] Hence, it seems that spatial know-how, as recruited by reaching, pointing and grasping movements, is neither necessary nor sufficient for bodily experiences. (2011, p. 200)

More generally, the empirical results described have been taken as evidence of a division between representations for conscious bodily awareness and representations for action, or between a somatosensory and proprioceptive processing stream for conscious bodily awareness and a somatosensory and proprioceptive processing stream for action (Dijkerman and de Haan 2007; Wong 2017).^7^ As with the Two Visual Systems Hypothesis, this seems to pose a threat to the claim that the spatial content of bodily awareness depends constitutively on the perceiver’s bodily action. Indeed, De Vignemont concludes that:

[…] empirical results both from neuropsychology and psychology […] show that (i) there are at least two kinds of bodily spatial contents, (ii) action determines only one of them, and (iii) the one determined by action is not the one used in conscious bodily experiences. (2009, p. 101)

## Resisting the standard explanation

I believe that de Vignemont’s conclusion can be challenged. It can be challenged because it requires that we assume that we must account for the difference in performance in the pairs of tasks employed in the empirical work in terms of differences in information, or representation. But, I will argue, there are two possible explanations of the difference in performance on each pair of tasks. The standard explanation accounts for the difference in performance in terms of two separate processing streams: one stream processing information about the body for conscious perception, the other stream processing information about the body for action. But according to an alternative explanation, which I will develop below, differences in performance can be accounted for in terms of (selective) shifts in mapping between different reference frames. And, as I will try to show, this alternative explanation is consistent with the Dispositional View.

I’ll focus here on the study into KE’s competence in locating tactile stimulation with pointing tasks, and Kammers et al.’s study using the RHI. In both studies, subjects are given two tasks.

In the study into the RHI, only one task requires the subject to produce a pointing response. The inter-manual pointing task assessed their capacity to reach with one hand to the location of the index finger on the other hand, without being able to see either hand (or the rubber hand).

In the proprioceptive drift task, a board was placed over the rubber hand and participant’s own hands, occluding all three of them. The experimenter then ran their index fingers along the board, starting either from the midline or the edges of the board, and the participant was asked to indicate when the experimenter’s fingers, which they could see, mirrored the perceived position of their own unseen index fingers.^8^ Participants, therefore, are asked to match a ‘felt’ location with a ‘visual’ location: that is, they are asked to pick out the felt location of their unseen hand by reference to the location of something they can see.

While subjects are able to point accurately to one index finger with the other hand in the inter-manual pointing task, they mislocate their hand in the proprioceptive drift task. This difference in task performance is explained on the standard explanation in terms of a difference in body representations. The proprioceptive drift task is taken to reflect conscious bodily awareness experience. Since participants mislocate their hand in this proprioceptive drift task, but perform the inter-manual pointing task accurately, it looks as though the content of conscious bodily awareness must be at odds with the proprioceptive information guiding action.

In the study of KE’s (and JO’s) capacities, the subjects were asked to perform two pointing tasks. In the inter-manual pointing task, subjects pointed with one hand to the other hand in the absence of vision. But rather than being asked to point to the tip of their index fingers, the subjects were asked to point with the unstimulated hand to the location on their other hand on which tactile stimulation had been delivered.

The second task, which I’ll call the cross-modal pointing task, was to point with their unstimulated hand to the location of stimulation on a diagram of the stimulated hand. In this task, subjects were able to see their unstimulated hand and the diagram they were to use to localize the sensation. So, here they had to identify a seen location (on the diagram) with a felt location and then produce a visuomotor response. Subjects have to pick out a location that they feel by selecting a location, on a diagram of the body part, that they can see.

The combination of KE’s failures to point accurately to the location of stimulation on his own hand in the inter-manual pointing task, and his relative success in pointing accurately to the location of stimulation on the diagram of his hand is taken to show that there must be two distinct processing systems for bodily awareness: a processing stream for conscious perception and a processing stream for action.

The standard explanation assumes that differences in task performance in both studies should be accounted for in terms of differences in representation. The different responses in the pairs of tasks must be explained in terms of a difference in proprioceptive or tactile information. But this overlooks another possibility: that what we have are cases in which there is (selective) recalibration of the mapping between different reference frames.

### Recalibration of the mapping between reference frames

In early sensory processing information about location is encoded in different, receptor-specific reference frames in the different senses. Vision initially encodes information about location in an eye-centred reference frame. Audition encodes spatial information in a head-centred reference frame. Touch encodes spatial information in multiple body-part-centred somatotopic reference frames (Deneve and Pouget 2004).

Any task involving sensory information about location from more than one sense will depend on there being a mechanism or system in place that allows for translation or mapping between these reference frames. To identify a place represented in one reference frame with a place represented in another reference frame, the two reference frames have to be calibrated relative to one another. Calibration between reference frames allows the perceptual systems to remap positions from one reference frame to the other. So, for example, in order to make a judgement about where my unseen hand is relative to something I can see—an experimenter’s hand, say—the perceptual systems must have some way of mapping between the visual reference frame and the proprioceptive reference frame.

Mapping between different sensory frames of reference depends on relationships that may change over time. Some of these changes will take place over longer periods of time and will be gradual. For example, the spatial relationship between the eyes and the ears changes somewhat as the body grows in childhood and adolescence. Other changes might be swift and short-lived: the spatial relationship between the eyes and each of the hands is subject to rapid and frequent change. Because remapping from one frame of reference into another depends on relationships that change over time, the alignment of frames of reference cannot be hard-wired. When reference frames become misaligned, the mapping between them must be subject to recalibration.

Recalibration is not limited to the mapping between sensory reference frames. In order for us to localize a target that we can see, information about the spatial location of the target that is encoded in vision must be mapped into a frame of reference suitable for generating the response. If the response required involves movement—pointing or looking, for example—information must be mapped into a frame of reference suitable for generating a motor response. The response will depend on the way information about location encoded in different sensory modalities maps onto motor outputs. And, as with the mappings between sensory reference frames, the mapping from a sensory reference frame to motor outputs must also be subject to recalibration.

### Explaining proprioceptive drift in terms of recalibration

There might be a number of ways in which the perceptual systems register misalignment between reference frames. One possibility is that synchronous but spatially discrepant stimulation may be sufficient to indicate an error in the current mapping, and so lead to recalibration.

Say that I see a hand being stroked and simultaneously feel my hand being stroked. The simultaneity provides evidence to the perceptual systems that there is a single stroking event. However, according to the current mapping between the visual reference frame and the tactile reference frame, what I see and what I feel are not at the same location. The simultaneity of the seen and felt stroking in combination with this spatial discrepancy may be interpreted by the perceptual systems as evidence of an error in the mapping between reference frames. This might then prompt recalibration of the mapping so that the location of what I see in the visual reference frame is identified with the location of what I feel in the tactile reference frame.^9^

In the rubber hand illusion the subject’s hand and the rubber hand are stroked in the same way with the same kind of object and with a similar onset and offset of stroking. This may be sufficient to indicate to the sensory systems that there is a single stroking event.^10^ However, the rubber hand and actual hand are at different locations. Assuming that the current calibration of the visual, tactile and proprioceptive reference frames is roughly correct, the location of the rubber hand in the visual reference frame will not correspond to the location of the actual hand in the tactile and proprioceptive reference frames. This may be sufficient to act as an indication of error in the mapping, and to instigate recalibration such that the location of the hands in each reference frame come to be mapped to one another. The location of the rubber hand in the visual reference frame comes to be identified with the location of the actual hand in the tactile and proprioceptive reference frames.

This kind of erroneous recalibration between vision and proprioception can explain subjects’ judgements in the proprioceptive drift task. Participants are asked to match a felt location with a visual location (either a value on a ruler or the position of the experimenters two index fingers). If the mapping between visual and proprioceptive reference frames has been recalibrated in the way I have suggested, when subjects try to indicate where their hand is by selecting a visual location, they will select a visual location that is closer to the location of the rubber hand. So, recalibration of the mapping between reference frames in the manner I have suggested can explain the biasing effect found in proprioceptive drift.

And, importantly, as long as recalibration is limited to the mapping between proprioception and vision, and doesn’t impact the mapping between proprioceptive inputs and motor outputs, it won’t produce any changes in where subjects attempt to point to when they are asked to perform the inter-manual pointing task. We therefore have an explanation of the difference in task performance in Kammers et al.’s study of the RHI that doesn’t require that we posit two distinct somatosensory processing streams, processing different information about the location of the hand.

### Explaining KE’s performance in terms of recalibration

KE’s task performance might also be explained in terms of recalibration of the mapping between reference frames. But KE manages with relative success to perform the cross-modal task he is asked to perform: when asked to point to the location that has been stimulated on a diagram of his hand, KE is fairly accurate. So, we shouldn’t posit a recalibration of the mapping between somatosensory and visual reference frames in the case of KE. What KE isn’t so good at doing is locating the point of stimulation on his own hand. It is, I suggest, possible to explain his failures on this task in terms of a shift in the mapping between tactile inputs and motor outputs. The result will be that tactile stimulation at location L_1_ on his hand will generate a motor output that results in a pointing action to, say, location L_3_ on the hand.

As with the RHI, as long as we allow that the connections between tactile inputs and motor outputs can change without there being any recalibration of the mapping between tactile and visual reference frames, then we can account for KE’s performance across the two tasks in terms of selective recalibration. And again, what the recalibration account avoids doing is positing two distinct streams of somatosensory processing, each one processing different information about the location of tactile stimulation.

### Evidence of recalibration of the mapping between reference frames

We have empirical evidence that indicates that recalibration of the mapping between reference frames takes place. The crossmodal congruency effect is a multisensory version of Posner’s spatial cueing paradigm, in which subjects are presented with two stimuli—a cue and a target—one after the other. So, for example, vibro-tactile stimulation on the hand might be followed by a light flash either near the same hand or near the subject’s other hand. Tactile stimulation of one hand results in faster and more accurate judgements about the elevation of a visual stimulus if it is presented from the same side (Driver and Spence 1998a).^11^ Here the tactile cue attracts attention to a region of space so that a visual stimulus that is presented close to the location of tactile stimulation will be processed more quickly than one presented in the other hemispace. For the selection of attention to be directed to locations or regions of space across vision and touch in this way, there must be a mechanism that allows for mapping between visual and tactile reference frames.

But, importantly, there’s a reversal of the cross-modal cueing effect after significant changes in posture. Driver and Spence (1998a) asked subjects to perform the crossmodal cueing task in two conditions: one in which their arms and hands were uncrossed; and one in which their arms and hands were crossed across the midline, so that the left hand was in right hemispace and the right hand in left hemispace. They found that the crossmodal congruency effect was now reversed: a visual flash on the left side produced faster up/down responses to tactile stimulation on the right hand, which was now positioned in left hemispace. As Driver and Spence say, “Evidently, the spatial mapping from particular retinal activations in vision, to somatoptopic activations in touch, gets updated when the hands adopt different postures.” (1998a, p. 1322).^12^

Visuomotor learning is by now a well-established research field, inspired by Hermann von Helmholtz’s discovery of prism adaptation. Von Helmholtz (1866) found that when he wore prism spectacles that displaced the visual field laterally to the left he first systematically over-reached when attempting to point to visual targets. After repeated attempts to point to the target his performance improved, but, when Helmholtz then removed the prism spectacles and attempted to point to a visual target, he now pointed too far to the right. The effect and aftereffect, much studied since Helmholtz made his observations, can be explained in terms of the recalibration of the mapping between visual and motor frames of reference.

Putting on prism spectacles establishes a misalignment between visual and motor reference frames, resulting, in the first place, in mistakes in our attempts to point to the things that we see. When we see the mistakes we make in pointing, we are able to then correct our pointing responses. Detecting this error and correcting for it brings about a recalibration of the mapping between the visual and motor frames of reference. The result of this recalibration is that, when the prisms are removed and normal vision is restored, there is a second temporary misalignment of visual and motor frames of reference in the opposite direction.

What I’ve offered here is an alternative explanation of the subjects’ performance in the tasks used in studies of the RHI, and studies of the capacities of patient KE. It’s an explanation that, if correct, would undermine the claim that the RHI or the neurological damage sustained by KE provide support for a functional distinction between proprioception for perception and proprioception for action. What I have tried to do, therefore, is call into question a central assumption underlying the claim that we have found dissociations between bodily awareness for perception and for action. It is possible to explain differences in task performance in terms of two distinct representations of the body, but it’s also possible to explain differences in task performance in terms of selective recalibration of the mapping between reference frames. And while the standard account, in terms of two distinct representations of the body, may seem to offer the simpler explanation of the empirical results, I think this is not in fact the case.

The tasks that have been used in studies that have been taken as providing evidence of a double dissociation between bodily awareness and action require mapping, either between sensory reference frames, or from sensory inputs to motor outputs. How subjects respond in each task will depend on the current mapping in question. And, importantly, the different tasks involve mapping between different reference frames. These are things that both the proponent of the standard account and I should agree on. What is more, we have evidence that shows that the mapping between different reference frames must be subject to recalibration. So, my opponent should concede the existence of mechanisms allowing for recalibration of the mapping between reference frames. But this is all that I require to explain the dissociation in performance on the two kinds of localization tasks discussed above. By contrast, the proponent of the standard explanation argues that we must, in addition to this, posit the existence of two distinct representations of the body, which must be maintained in parallel to one another, and which represent at least some of the same properties of the body. So while the explanation given may appear to be simpler because it can be stated more concisely, the ontological commitments it makes look to be more onerous.

What is more, by accounting for the difference in performance across the pairs of localization tasks in terms of recalibration, we offer an explanation of this particular set of experimental results that falls within a much more general account of the kind of mechanisms that must be in place to deal with sensory processing across different sensory systems in which spatial information is encoded in different formats, and which we need to appeal to in order to explain a wide range of behavioural effects involving vision, audition, touch and proprioception, as indicated above.

Considerations of parsimony and generality are not enough to show that my alternative is the correct explanation of performance across the pairs of tasks. So, I haven’t offered what amounts to a defense of the Dispositional View of the spatial content of bodily awareness. But, I suggest, these considerations do give us reason to take the alternative account seriously. What we need, then, is further argument, or more empirical work to determine which explanation is the correct one.

## Conclusion

The Dispositional View of the spatial content of bodily awareness has come under pressure from a series of empirical results that have standardly been taken to show that there is a functional distinction between processing of proprioceptive and tactile information for perception and processing of proprioceptive and tactile information for action.

I have tried to argue that the standard explanation of the empirical work underpinning this rests on assumptions about the kinds of tasks that enable us to measure where subjects feel parts of their body and bodily sensations to be, and where we must look for an explanation of differences in the performance of different kinds of task.

I have suggested there is an alternative explanation of why it is that subjects locate or mislocate body parts or particular sensations in particular tasks. I have proposed that participants in the RHI do not have bodily awareness experiences with illusory spatial content. What the alternative explanation of the RHI does, in effect, is suggest that motor behaviour and bodily awareness can be brought into line with one another. The alternative explanation of the capacities and deficits that KE exhibits focuses on the mapping between visual and tactile reference frames and the mapping between tactile inputs and motor outputs. It suggests that the former can be preserved when the established connections between tactile inputs and motor outputs have been disrupted.

Overall, my claim is that the tasks that have been used to assess bodily awareness require mapping between different reference frames. Crucially, though, the mapping required for each task is different. Some tasks require mapping between sensory reference frames (proprioceptive drift task), other tasks require mapping from a sensory reference frame to a motor output (inter-manual pointing task), and some require a combination of the two (cross-modal pointing task). So, I have argued, its possible to account for the difference in performance across the two tasks in each study in terms of changes or breakdowns in certain mappings, but not others. Therefore, the behavioural results, I have tried to suggest, don’t allow us to determine whether bodily awareness and action can come apart or not. This doesn’t, of course, amount to a defence of the Dispositional View. But I think it indicates the need for further work to establish whether or not we have evidence of dissociations between conscious bodily awareness and bodily action, and so whether or not we have evidence that undermines the Dispositional View.

## Footnotes

1 This is far from an a exhaustive list of the information available through the body senses, which should also include at the least information about the state of various internal organs required for homeostatis, and information about balance and orientation from the vestibular system in the inner ear.

2 ‘Bodily awareness’ may be used to pick out additional kinds of experience of our bodies ‘from the inside’, including feelings of tiredness and hunger, an awareness of one’s heartbeat, and so on. In this paper my focus is on the spatial content of bodily awareness. To the extent that these bodily sensations have a felt location—and I would submit that they do—I would seek to extend the account defended in this paper to the spatial content of those experiences also. For the sake of exposition, I will, for the most part, limit my discussion to our experience of tactile sensations and bodily posture.

3 There are, of course, cases in which our bodily sensations have a somewhat diffuse or indeterminate spatial phenomenology (Smith 2009). We might say, then, that there is a range of spatial determinacy in bodily awareness. Some bodily sensations have a more determinate felt location—a stinging sensation produced by a mosquito bite, say—while others have a less determinate felt location—an ache in one’s thigh, perhaps. Still, both sensations have an apparent location.

4 Briscoe (2009), while focusing on egocentric spatial representation in vision, suggests that we can apply Evans’s account of egocentric spatial content to proprioception.

5 In another approach, Smith (2009) suggests that bodily locations are given in bodily awareness in terms of the actions that the body part in which the sensation is felt to be located affords. See de Vignemont (2015) for a critique of this proposal.

6 De Vignemont (2009, 2011, 2018) argues that we should take numbsense to be a case in which spatial know-how is preserved in the absence of conscious tactile and proprioceptive experience, and so that, “the case of numbsense therefore shows that spatial know-how (and its exercise) per se is not a sufficient condition of tactile and proprioceptive phenomenology” (2011, p. 197).

7 The apparent dissociations between bodily awareness and action have often been presented in the literature as evidence of two distinct kinds of body representation: the body schema and the body image (Paillard 1999; Gallagher 2005), where, “The body image is a representation of one’s overall body form that can be manifest in consciousness through perception or imagery. It is an explicit representation of body form, which may include the conscious awareness of current postural configuration. In contrast, the body schema is a dynamic representation of the relative position of body parts that is employed in the control of action and the maintenance of posture. It is typically unconscious” (Wong 2017, p. 8). So, for example, numbsense has been understood to be a case in which the body schema is intact, while the body image is compromised (Paillard 1999), while peripheral proprioceptive deafferentation has been interpreted as a case in which the body image is intact while the body schema is absent (Cole and Paillard 1995; Paillard 1999). However, the exhaustive categorisation of representations of the body into body image and body schema is controversial (for a critique see, for example, de Vignemont 2010).

8 In many studies on the RHI (for example, Tsakaris and Haggard 2005) proprioceptive drift is measured using a ruler, which is placed on top of a visual divider that occludes the subjects own hands and the rubber hand. The location of the index finger is judged by selecting a value on the ruler. As in Kammers et al.’s study, this requires subjects to pick out the location at which they feel their hand to be by reference to something they can see.

9 Recalibration might at times be deficient or incomplete, so a location in a visual reference frame comes to be identified with a location in a tactile reference frame that is at the mid-point between the location it was previously identified with and the location of the object in that reference frame that instigated the recalibration. The detection of error resulting in recalibration might also be mistaken, so that recalibration should not but does occur. This is what I am suggesting occurs in the rubber hand illusion.

10 While temporal coincidence is considered to be a factor in whether or not multisensory interactions take place, there is evidence that there is a temporal window in which stimulation in two different modalities must occur in order for an interaction to take place, so strict temporal synchrony is not in fact required (see, for example, Spence and Squire 2003).

11 The same is true when the modality of cue and target is switched round: a visual flash presented prior to and near to tactile stimulation of the hand results in quicker and more accurate tactile judgements (Spence and Driver 1997; see Driver and Spence 1998b also).

12 Similar results after postural change are found in the crossmodal congruency effect (Spence et al. 1998; Pavani et al. 2000). Deneve and Pouget also cite results from patients with neglect as providing further evidence of crossmodal spatial attentional effects (2004, p. 253).

## References

[CR1] Anema HA, van Zandvoort MJE, de Haan EHF, Kappelle LJ, de Kort PLM, Jansen BPW, Dijkerman HC (2009). A double dissociation between somatosensory processing for perception and action. Neuropsychologia.

[CR2] Bermúdez JL, de Vignemont F, Alsmith A (2017). Ownership and the space of the body. The subject’s matter: Self-consciousness and the body.

[CR3] Botvinick M, Cohen J (1998). Rubber hands “feel” touch that eyes see. Nature.

[CR4] Brewer B, Bermúdez JL, Marcel A, Eilan N (1995). Bodily awareness and the self. The body and the self.

[CR5] Briscoe R (2009). Egocentric spatial representation in action and perception. Philosophy and Phenomenological Research.

[CR6] Briscoe R (2014). Spatial content and motoric significance. AVANT.

[CR7] Clark A (1999). Visual awareness and visuomotor action. Journal of Consciousness Studies.

[CR8] Clark A (2001). Visual experience and motor action: Are the bonds too tight?. The Philosophical Review.

[CR9] Clark A (2007). What reaching teaches: Consciousness, control and the inner zombie. British Journal for the Philosophy of Science.

[CR10] Cole J, Paillard J, Bermúdez JL, Marcel A, Eilan N (1995). Living without touch and peripheral information about body position and movement. The body and the self.

[CR11] de Vignemont F (2009). Bodily spatial content. PSYCHE: An Interdisciplinary Journal of Research on Consciousness.

[CR12] de Vignemont F (2010). Body schema and body image–Pros and cons. Neuropsychologia.

[CR13] de Vignemont F (2011). A mosquito bite against the enactive approach to bodily experiences. Journal of Philosophy.

[CR14] de Vignemont F, Coello Y, Fischer MH (2015). Bodily affordances and bodily experiences. Perceptual and emotional embodiment.

[CR15] de Vignemont F (2018). Mind the body.

[CR16] Deneve S, Pouget A (2004). Bayesian multisensory integration and cross-modal spatial links. Journal of Physiology.

[CR17] Dijkerman HC, de Haan EHF (2007). Somatosensory processes subserving perception and action. Behavioral and Brain Sciences.

[CR18] Driver J, Spence C (1998). Cross-modal links in spatial attention. Philosophical Transactions of the Royal Society London B.

[CR19] Driver J, Spence C (1998). Attention and the cross-modal construction of space. Trends in Cognitive Sciences.

[CR20] Evans G (1982). The varieties of reference.

[CR21] Gallagher S (2005). How the body shapes the mind.

[CR22] Grush R (2007). Skill theory v2.0: Disposition, emulation, and spatial perception. Synthese.

[CR23] Hurley SL (1998). Consciousness in action.

[CR24] Kammers MPM, de Vignemont F, Verhagen L, Dijkerman HC (2009). The rubber hand in action. Neuropsychologia.

[CR25] Martin MGF, Bermúdez JL, Marcel A, Eilan N (1995). Bodily awareness: A sense of ownership. The body and the self.

[CR26] Noë A (2004). Action in perception.

[CR27] O’Shaughnessy B (1980). The will: A dual aspect theory.

[CR28] Paillard J, Gantchev GN, Mori S, Massion J (1999). Body schema and body image: A double dissociation in deafferented patients. Motor control, today and tomorrow.

[CR29] Paillard J, Michel F, Stelmach G (1983). Localization without content. A tactile analogue of ‘blindsight’. Archives of Neurology.

[CR30] Pavani F, Spence C, Driver J (2000). Visual capture of touch: Out-of-the-body experiences with rubber gloves. Psychological Science.

[CR31] Smith AJT (2009). Acting on (bodily) experience. PSYCHE: An Interdisciplinary Journal of Research on Consciousness.

[CR32] Spence C, Driver J (1997). Audiovisual links in exogenous covert spatial orienting. Perception and Psychophysics.

[CR33] Spence C, Pavani F, Driver J (1998). What crossing the hands can reveal about visuotactile links in spatial attention. Abstract of the Psychonomic Society.

[CR34] Spence C, Squire S (2003). Multisensory integration: Maintaining the perception of synchrony. Current Biology.

[CR35] Tsakaris M, Haggard P (2005). The rubber hand illusion revisited: Visuotactile integration and self-attribution. Journal of Experimental Psychology: Human Perception and Performance.

[CR36] von Helmholtz, H. (1962). 1866, *Treatise on physiological optics* (Vol. 3). English translation of 3rd German edition. New York: Dover.

[CR37] Wong HY, Coello Y, Fischer MH (2015). The body schema as a condition of possibility for action. Perceptual and emotional embodiment.

[CR38] Wong HY (2017). Embodied agency. Philosophy and Phenomenological Research.

